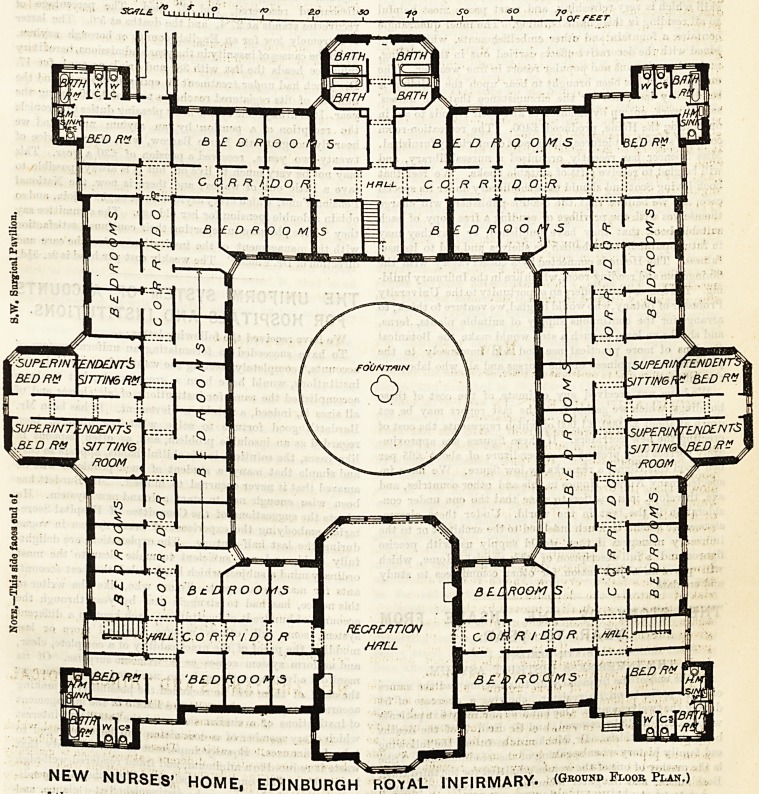# Nurses' Home, Edinburgh Royal Infirmary

**Published:** 1893-11-25

**Authors:** 


### The Institutional Workshop.

### HOSPITAL CONSTRUCTION.

### NURSES' HOME, EDINBURGH ROYAL INFIRMARY.

The erection of this Home marks a new and important departure in the history of the Royal Infirmary. Until it was opened and occupied, although the new Infirmary was one of the most complete of the multiple pavilion hospitals of the world, from an absence of adequate accommodation for the nurses it was difficult to maintain them in health, and, what was almost as bad, the staff of nurses was not large enough to provide a maximum of care and attention to the large number of patients for whom ward accommodation has been built. The new Home has enabled the managers to adequately increase the number of the nursing staff, and it has placed the nursing staff itself, so far as health and recreation are concerned, under the most favourable hygienic conditions. The Home stands in the centre of the Infirmary grounds, and is a strikingly handsome building considering that the architect, Mr. Sydney Mitchell, was hampered by the necessity of keeping the buildings as low as possible. Accommodation has been provided for one hundred and twenty-one nurses, in addition to a spacious recreation-room and a series of sick-rooms, which are so excellent as to cheer the heart of every intelligent administrator who visits them.

The ground floor plan (which we publish to-day) will afford a good idea of the scheme of this, the latest and by far the best Nurses' Home yet erected. It will be seen that each nurse is provided with a bed-room, and that the Superintendents have

NEW NURSES' HOME, EDINBURGH ROYAL INFIRMARY. (Ground Floor Plan.)

suites of rooms for their accommodation. The bed-room vary in size and shape, which gives variety and has afforded scope for decorative effect which might otherwise have been wanting, every bed-room is self-contained as to its furniture, ventilation, and heating, and in the decoration of the Home much excellent taste has been exhibited. We have seen few prettier effects than those presented by the corridors on the first floor, whilst the colour and tone of the decorations exhibit an amount of artistic skill which is very refreshing, and must prove most helpful to all residing in this chaste building. The inner quadrangle contains a fountain and other embellishments, which, combined with the decorative effects carried out in the building, makes it a pleasant and popular resort in fine weather. The energy which has been brought to bear upon the building is sufficiently indicated by the circumstance that the nurses' sale of work, got up by themselves and their friends to aid in decorating the Home, produced £400. The recreation-room contains some fine pictures, and is most comfortably furnished. Miss Spencer has recently organised a nurses' library, and will be glad to receive gifts of suitable books. We feel that book-loving Scotland should take the library under its special care, and we believe that the Scotch publishers will charge themselves with the privilege of sending a free copy of each suitable book that they have published, or that they may in future publish, to embellish its shelves and add to its usefulness. The Home is connected by a conservatory with the diningroom and reading-room, which are in the Infirmary building. The conservatory offers an opportunity to the University Professor of Botany, who would be glad, we venture to think, to arrange for the continuous supply of suitable plants, ferns, and shrubs on loan, as such a step would make the Botanical Gardens of more practical use and add immensely to the pleasure and happiness of the nurses and all who labour in the Infirmary.

We have not received any estimate of the cost of these buildings, which we gather from the last report may be set down at about £8,000, £1,000 of which represents the cost of erecting the new corridors. If these figures are approximately correct they present an expenditure of about £65 per bed upon this Home, a remarkably low figure. We have inspected many Nurses' Homes in this and other countries, and are, therefore, in a position to state that the one under consideration is the best in the world. Under these circumstances we should be much indebted to the architect or to the infirmary managers if they would supply us with precise figures and a full description of this special Home, which will prove an object lesson for other committees to study and emulate.

## Figures and Tables

**Figure f1:**